# 2018 Nobel Prize in physiology or medicine

**DOI:** 10.1002/cti2.1041

**Published:** 2018-10-24

**Authors:** Mark J Smyth, Michele WL Teng

**Affiliations:** ^1^ Immunology in Cancer and Infection Laboratory QIMR Berghofer Medical Research Institute 300 Herston Road Herston 4006 QLD Australia; ^2^ Cancer Immunoregulation and Immunotherapy QIMR Berghofer Medical Research Institute 300 Herston Road Herston 4006 QLD Australia

## Abstract

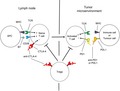

American James Allison (University of Texas MD Anderson Cancer Center) and Japan's Tasuku Honjo (Kyoto University School of Medicine) have won the 2018 Nobel Prize for Physiology or Medicine for discoveries leading to new approaches in harnessing the immune system to fight cancer. This award is particularly special for those, like us, who have spent their careers with the belief that the immune system could impact on cancer initiation, growth and spread.^1^ Even though the clinical value of immunotherapy is now widely accepted, for many years Dr Allison and others, most notably Dr Lloyd Old, had to lead and convince many scientists, medical oncologists and pharmaceutical companies to believe in and invest in immune‐based approaches, which were considered of little merit by mainstream oncology. In Australia, their leadership remained an inspiration for the small group of dedicated tumor immunologists that existed through those times. Following *Science*'s announcement of immunotherapy as the Breakthrough of the Year in 2013, this Nobel Prize represents the latest validation of the benefits borne by immunotherapy's progress over the past few decades.

For 100 years or more, cancer treatment has been dominated by surgery, radiation and chemotherapy, including various targeted therapies. But immunotherapy, paradigm shifting by virtue of targeting the host immune system rather than cancer cells themselves, now takes its place as a major armamentarium in the war on cancer. Over the past decade, immune checkpoint inhibitor (ICI) immunotherapies that target cytotoxic T‐lymphocyte‐associated protein 4 (CTLA‐4) or the programmed cell death protein 1 (PD‐1) pathway have achieved impressive success in the treatment of different cancer types. These two ICIs, which target inhibitory receptors on T cells and reinvigorate antitumor immune responses (Figure 1), have begun to transform clinical cancer care. In 2010, the humanised anti‐cytotoxic T‐lymphocyte antigen 4 (CTLA‐4) antibody, ipilimumab, became the first treatment of any type to improve survival in metastatic melanoma patients, doubling 10‐year survival rates for metastatic melanoma compared to historical data.^2^, ^3^ In 2011, ipilimumab was approved by the US Food and Drug Administration (FDA) for clinical use in advanced melanoma, which marked a turning point for immunotherapy.

**Figure 1:**
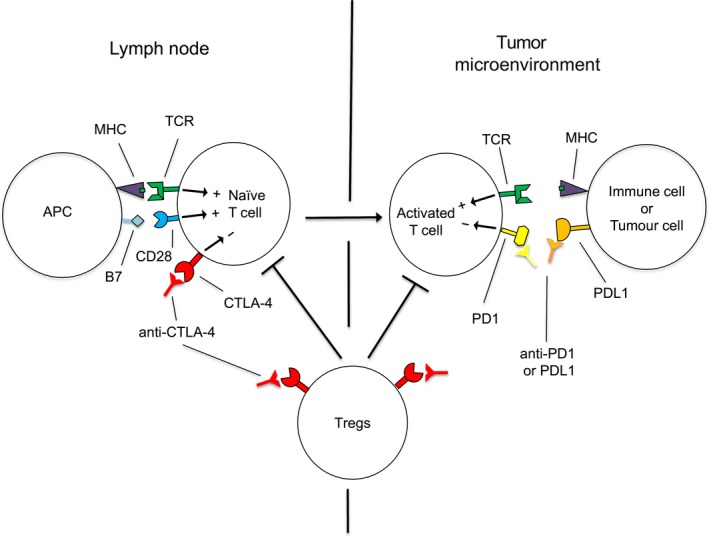
Antibodies that block CTLA‐4 or PD‐1/PD‐L1 can induce antitumor responses. (left), Following the initial activation of a tumor‐specific T cell in the lymph node with antigen‐presenting cells (APC) through the interaction of the T‐cell receptor (TCR) with MHC presented tumor‐derived peptide, CTLA‐4 is upregulated and acts as a negative regulator of costimulation, which can be blocked with anti‐CTLA‐4 antibodies. Blockade of CTLA‐4 on regulatory T cells (Tregs) also relieves their suppression of T‐cell activation or effector function. (Right), Activated T cells circulate throughout the blood and traffic into tumors where they can be activated upon recognition of cognate antigen presented by cancer or immune cells. This activation leads to upregulation of PD1 and production of IFN‐γ, which can upregulate PD‐L1 on both tumor and immune cells in the tumor microenvironment. This mechanism, termed adaptive immune resistance, can suppress effector function of tumor‐specific T cells, which can be re‐invigorated using antibodies that block PD1 or PD‐L1.

Blockade of another immune checkpoint molecule, programmed cell death 1 (PD‐1), or its ligand, PD‐1 ligand 1 (PD‐L1), was shown to provide a survival advantage in a number of different malignancies, with higher response rates and lower incidence of side effects compared to anti‐CTLA‐4.^4^, ^5^, ^6^, ^7^, ^8^, ^9^ Accordingly, antibodies targeting the PD‐1:PD‐L1 axis have been approved as second‐ or first‐line therapies for an ever‐growing list of malignancies, including melanoma, lymphoma, lung cancer, renal cancer, head and neck cancer, bladder cancer, liver cancer, gastroesophageal cancer and cutaneous squamous cell carcinoma.^10^ Six of these ICIs have now been approved by the FDA for various cancers, including one that became the first and only cancer treatment of any type to be approved for tumors with a certain genetic biomarker, regardless of their tissue of origin (i.e. cancer agnostic). The ultimate in confluence of these approaches came with the description of superior outcomes in advanced melanoma in patients receiving a combination of anti‐CTLA‐4 and anti‐PD‐1.^6^

This contemporary revolution in cancer treatment owes its success to the dedication and insight that James Allison and Tasuku Honjo displayed in their early studies on CTLA‐4 (1995) and PD‐1 (1992, 1999, 2000), respectively.^11^, ^12^, ^13^, ^14^, ^15^ Importantly, for immunology in general, they determined the immune checkpoint (‘brake’) function of these molecules in T cells, with distinct mechanisms of action. Despite some seminal functional studies with CTLA‐4‐Ig fusion proteins that demonstrated immunosuppression,^16^, ^17^ there was confusion about the functions of CTLA‐4 versus the costimulator CD28. The mechanistic definition that CTLA‐4 opposed CD28 in action by Krummel and Allison was a key breakthrough^11^ and the negative regulatory role of CTLA‐4 was revealed by gene targeting in mice.^18^, ^19^ The inhibitory effects of CTLA‐4 are critical for normal immune system function, serving as a ‘checkpoint’ that restricts the immune system from inappropriately attacking healthy self‐tissue. These findings were fundamental to understanding that these limiting self‐protective molecules expressed by T cells might hinder a patient's natural and therapy‐induced immune response to cancer. Establishing proof of concept, Dr Allison helped show that CTLA‐4 functions as a ‘brake’ on T cells and he was the first to demonstrate that blocking CTLA‐4 with an antibody could prevent tumor development in mice as well as enable them to eliminate large, established tumors.^20^ Later realised was the elevated expression of these immune checkpoint molecules in chronically activated T cells, including those within the tumor microenvironment. Initially driven to better understand how T cells function, Allison had additional motivation from his own family's encounters with cancer, to strive to translate his findings into medicine for human patients.

The Nobel Prize is the ultimate honour for Allison and Honjo who between them have careers littered with prizes and awards, including the William B. Coley Award (Allison in 2005 and Honjo in 2014), the Robert Koch Prize (Honjo in 2012), the inaugural Lloyd J Old Award (Allison in 2013), the Novartis Prize for Clinical Immunology (Allison in 2013), the Lasker‐DeBakey Clinical Medical Research Award (Allison in 2015) and the Tang Prize for Pharmaceutical Science (Allison/Honjo shared in 2014). Of course, these two great immunologists are recognised for so many other important discoveries, including the discovery of IL‐4, IL‐5 and activation‐induced cytidine deaminase (ACD) by Honjo and his critical work on class switching in B cells. Allison performed a significant body of work on the T‐cell receptor and CD28 costimulation in the 1980s and, to this day, each continues to be a leader and stellar contributor to the field of T‐cell immunology and its application to disease. Indeed, these two scientists have beautifully mixed biochemistry and immunology and employed the mouse as a preclinical model to uncover immune cell mechanism of action. Importantly, their work was not performed in isolation, but rather in the milieu of decades of work, performed in hundreds of laboratories, on the basic architecture and function of T cells and the immune system. In the absence of such fundamental research, Allison and Honjo's ground‐breaking work would never have been possible. Lastly, given recent and widespread scepticism about the value of mouse research, it is worth remembering that this prize, and the decades of work leading up to it, was not possible without extensive use of mouse tumor and immune deficiency models. They provided critical insight to define the checkpoint nature of CTLA‐4 and PD‐1 on T cells and the massive importance of both of these pathways to preventing effective immune response to cancer.

We may not have heard the last of Nobel recognition of cancer immunotherapy. Adoptive cellular T‐cell therapies pioneered by Steven Rosenberg, and the related chimeric antigen receptor (CAR) T‐cell approach, have also produced spectacular advances in the treatment of melanoma and blood cancers. Although currently less amenable to high clinical throughput and less broad in their cancer treatment utility, these and perhaps many other cancer immunology approaches will likely impact on patient outcomes. One of the most important recent advances concerns the earlier use of ICI in neoadjuvant therapy in the context of surgery – and here the first results look extremely promising.^21^, ^22^ Indeed, it remains very early in the implementation of cancer immunotherapy into mainstream oncology practice and one would envisage further improvements even with the existing arsenal of immunotherapies at hand.

Like all great discoveries, many questions have been raised, and indeed, it is fair to say that even the exact mechanism of action of these ICI antibodies targeting human CTLA‐4 or PD‐1 remains in dispute. However, regardless of mechanism of action, these ICI immunotherapies serve as wonderful positive controls in some patients in diseases where the patients previously had no significant life expectancy. These new immunotherapeutics developed in the context of high‐throughput gene sequencing have taken human oncology research into an era of tumor mutational burden and neoantigens and the search for predictive markers and the further development of personalised medicine. The oncology and immunology communities can together stand on the shoulders of two giants, such as Allison and Honjo, to make breakthroughs that will further transform medicine.

Ultimately, clinical use will be governed not just by the science but by feasibility and reproducibility in the ‘real‐world’ clinical setting, cost and investment to establish prospective validation. The ongoing intensive work to establish and understand biomarkers for ICI response prediction holds great promise for maximising patient benefit from these transformative therapies.

From the Australasian immunology and translational research community, we extend our sincere gratitude for all that Drs Allison and Honjo have done to advance the field of cancer immunotherapy and to save the lives of many cancer patients.
